# Micro-Prism Patterned Remote Phosphor Film for Enhanced Luminous Efficiency and Color Uniformity of Phosphor-Converted Light-Emitting Diodes

**DOI:** 10.3390/mi12091117

**Published:** 2021-09-17

**Authors:** Jiadong Yu, Shudong Yu, Ting Fu, Yong Tang

**Affiliations:** 1 Guangdong Provincial Key Laboratory of Technique and Equipment for Macromolecular Advanced Manufacturing, South China University of Technology (SCUT), Guangzhou 510640, China; meyujd2020@mail.scut.edu.cn (J.Y.); ytang@scut.edu.cn (Y.T.); 2 Hubei Key Laboratory of Mechanical Transmission and Manufacturing Engineering, Wuhan University of Science and Technology, Wuhan 430081, China; futing1234gh@wust.edu.cn; 3 National and Local Joint Engineering Research Center of Semiconductor Display and Optical Communication Devices, South China University of Technology (SCUT), Guangzhou 510640, China

**Keywords:** micro-prism, light-emitting diode, remote phosphor

## Abstract

In this work, we propose micro-prism patterned remote phosphor (RP) films to enhance both luminous efficiency and color uniformity (CU) of remote phosphor-converted light-emitting diodes (rpc-LEDs) simultaneously. On the incident surface of the RP film, one micro-prism film is used to extract backward light by double reflection. On the exit surface, the other micro-prism film is adopted to retain blue light inside the RP film, thus enhancing the phosphor excitation. Experimental results show that double prism-patterned RP (DP-RP) film configuration shows a luminous flux of 55.16 lm, which is 45.1% higher than that of RP film configuration at 300 mA. As regards the CU, the DP-RP film configuration reduces the angular CIE-x and CIE-y standard variations by 68% and 69.32%, respectively, compared with the pristine device. Moreover, the DP-RP film configuration shows excellent color stability under varying driving currents. Since micro-prism films can be easily fabricated by a roll-to-roll process, the micro-prism patterned RP film can be an alternative to a conventional RP layer to enable the practical application of rpc-LEDs.

## 1. Introduction

White light-emitting diodes (WLEDs) are very promising light sources in general lighting, automotive headlamp, and liquid-crystal backlight due to their high efficiency, long lifetime, compact size and environmental friendliness [1,2,3]. The most widely used configuration to generate white light is the combination of a blue GaN chip and down-converted yellow phosphor, namely phosphor-converted light-emitting diodes (pc-LEDs), which has excellent durability, low cost and easy scalability [4,5,6]. It is well known that luminous efficiency (LE) and color uniformity (CU) are two important indexes of pc-LEDs [7,8,9]. In a traditional LED package, phosphor-silicone mixture is directly dispensed on a GaN chip, which leads to a low light extraction efficiency of LEDs due to the backscattered issue [8]. To enhance the LE of pc-LEDs, phosphor layer is separated from the chip (namely remote phosphor scheme) to extract the backscattered light, thus reducing the amount of absorbed light by the LED chip [10,11]. To achieve backward light extraction, scattered photon extraction (SPE) was first developed by Narendran et al. to efficiently extract backward light of remote phosphor-converted LEDs (rpc-LEDs) and the experimental results showed an improvement of over 60% in the light efficiency compared with conventional pc-LEDs [12]. However, the SPE structure has a high correlated color temperature (CCT), which is not appropriate for lighting [13], and the CU is also unsatisfactory, caused by the concentrated blue radiation in the center. Various packaging designs, including diffused reflection cup [14], domed remote phosphor (RP) structure [15], and reassembled RP geometry [16] were proposed to enhance the LE of rpc-LEDs. However, those packaging structures suffer from compromised CU, thereby limiting their practical applications in lighting. Indeed, the inhomogeneous color distribution of rpc-LEDs is caused by the mismatching of Lambertian blue radiation and isotropic emission pattern of the converted yellow light. To enable the practical application of rpc-LEDs, many technologies have been adopted to improve their angle-dependent CU, such as patterned phosphor structure [17], freeform phosphor film [18], shaped phosphor layer [19], and multi-layer configurations [20]. However, those methods usually result in a noticeable light loss. Up until now, there has been a lack of approaches that can improve the LE and CU of rpc-LEDs simultaneously. To the best of our knowledge, a remote phosphor layer is usually cast into a thin film with a sub-millimeter thickness, which leaves sufficient room to be tailored with micro-structures on the film surfaces [21,22]. In this study, we proposed a micro-prism-integrated strategy to improve the LE and CU of rpc-LEDs. In detail, both surfaces of the RP film are patterned with micro-prism films, which are crossed orthogonally in the placing direction. The micro-prism film on the incident surface of the RP film serves as a light splitter to reduce the central blue-light intensity and elongate the blue light path; it is also a good backward light reflector, enabling the effective extraction of backward blue and yellow light. Additionally, the micro-prism film on the exit surface of the RP film can trap the exiting blue light for enhanced absorption by the phosphor and assist in extracting the total reflected light into free space. Both micro-prism films are harnessed to enhance the LE and CU of rpc-LEDs. Therefore, the integration of micro-prism films provides a promising route to enable the practical applications of rpc-LEDs.

## 2. Materials and Methods

### 2.1. Experiment Materials and Methods

In the RP film preparation, polydimethylsiloxane (PDMS, Dow Corning Sylgard-184) was chosen as the phosphor host due to its high transparency, flexibility and good replication capability. Its mixture with yellow Y_3_Al_5_O_12_:Ce^3+^ (YAG: Ce^3+^) phosphor (YAG04, Intematix) was prepared at a weight ratio of 4:1, followed by the mixing with PDMS curing agent at a mass ratio of 10:1. The mixing process was conducted under vacuum to avoid the generation of air bubbles. Afterward, the phosphor-PDMS mixture was injected into a sealed mold and cured at 120 ℃ for 30 min to obtain a RP film with a uniform thickness of 300 μm.

**Figure 1:**
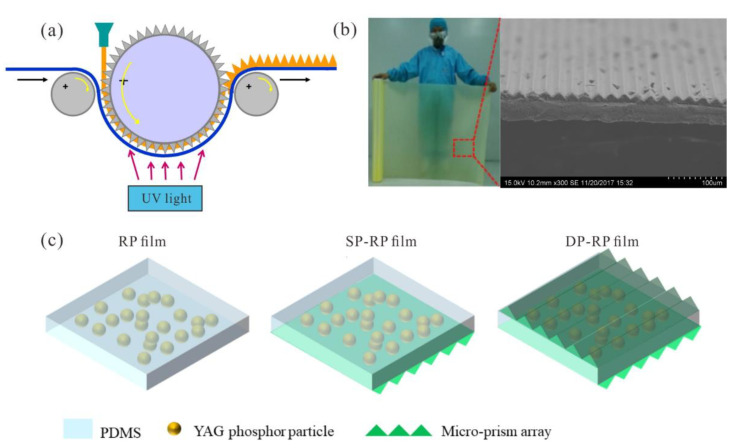
(**a**) Schematic diagram of roll-to-roll imprinting process; (**b**) the fabricated micro-prism film and its microstructure on the surface; (**c**) schematic diagram of three kinds of remote phosphor (RP) film, single-prism-patterned (SP-RP) film and double-prism patterned (DP-RP) film.

To fabricate micro-prism films, a roll-to-roll imprinting process was conducted on a commercial machine (A-Lumen), as shown in Figure 1a. Firstly, a master roll with micro-prism structure was fabricated by an ultra-precision drum lathe (ULR-628B(H), Toshiba). Polyethylene terephthalate (PET) membrane with a thickness of 38 μm (Toray, Tokyo, Japan) was selected as the substrate of an imprinted micro-prism array. Second, the PET film coated with liquid UV resin (80543A, Eternal Materials) was embossed and further cured under UV irradiation to generate a micro-prism array. Lastly, the micro-prism film was collected by the winding part of the imprinting machine. As shown in Figure 1b, the micro-prism film has a width of 1080 mm and the linear micro-prism structure was replicated well. The prism has an apex angle of 90°, a height of 12 μm and its pitch is 24 μm. We note that such micro-prism array is engineered to generate a double reflection effect when light is incident on the prism surface in the normal direction, which is assumed to effectively extract backward propagating light in the rpc-LEDs. The dimensions of the micro-prism were carefully selected to induce such a significant double reflection effect in the geometric optics regime. When micro-structures possess size comparable to the wavelength scale, they can lead to prominent scattering due to the diffraction effect as re-ported in our previous paper [8], which is detrimental to the double reflection effect. In addition, a micro-prism array with larger size has more ineffective optical zones in the gaps of prisms, which might deteriorate the double reflection effect. Furthermore, by taking account of the fabrication capacity of roll-to-roll process, we targeted a micro-prism array with a height of 12 μm and a pitch of 24 μm.

To obtain a patterned RP film, the fabricated micro-prism film was coupled with the planar RP film by virtue of the sticky nature of PDMS with the PET substrate of micro-prism film; hence a single-prism-patterned RP (SP-RP) film and a double-prism patterned RP (DP-RP) film were obtained, which are shown in Figure 1c. As regards the DP-RP film, we arranged two micro-prism films in an orthogonal direction to circumvent the redirection of blue light in the same direction twice, which is conducive to achieving homogenous blue light distribution in the free space. In addition, the rpc-LED device configuration contained a 3535-type LED component (Foshan Nationstar), a sleeve, a total internal reflection (TIR) lens and an RP film integrated on the exit surface of the TIR lens surface. The TIR lens had a height of 7 mm and its top surface diameter was 12 mm. The phosphor film was separated by the TIR lens to form a RP configuration.

### 2.2. Characterization Methods

The micro-prism structure was characterized by scanning electron microscopy (Merlin, Zeiss, Oberkochen, Germany). The radiant power, luminous flux and spectra were measured in a 0.5 m-diameter integrating sphere integrated with a spectrometer (LE5400, Otsuka, Tokyo, Japan). The LED devices were powered by a direct-current power source (Keithley 2450DC, Keithley Instruments, Solon, OH, USA) and the measuring currents were varied from 100 mA to 500 mA, corresponding to electrical powers changing from 0.27 W to 1.59 W. The angle-dependent CCT distributions at 300 mA were measured by a homemade instrument equipped with an Ocean Optics spectrometer positioned at a distance of 316 mm (CIE test method condition A).

### 2.3. Simulation Method 

To conduct ray tracing simulation, commercial software Tracepro was used in this work. In the simulation model, a grid light source with specific incident angle was used as the incident light source. Furthermore, the refractive index of film medium was set as ~1.5 without considering the chromatic dispersion phenomenon for the simplicity of the optical simulation. To introduce the micro-prism array into the optical models, a built-in reptile function was used instead of directly constructing structural micro-prism elements. After the ray tracing, a ray transmitting path was visualized to clarify the optical effect of both planar film and micro-prism film.

## 3. Results and Discussion

### 3.1. Optical Eeffects of the Micro-Prism Films on the Remote Phosphor-Converted Light-Emitting Diodes (Rpc-LEDs)

**Figure 2:**
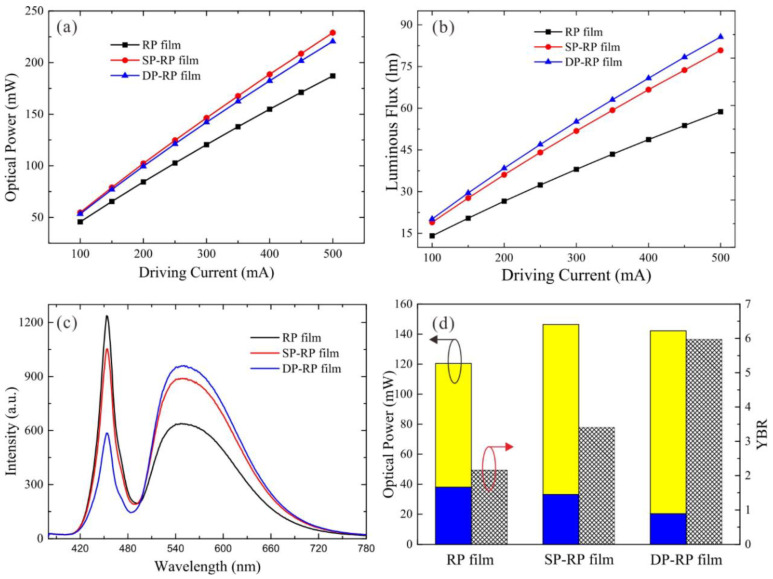
(**a**) Optical power and (**b**) luminous flux of the three remote phosphor-converted light-emitting diode (rpc-LED) devices at varying driving currents from 100 mA to 500 mA; (**c**) emission spectra of three rpc-LED devices at 300 mA and (**d**) their blue, yellow power and yellow-blue ratio (YBR) values.

Figure 2a,b shows the current-dependent optical power and luminous flux of the three rpc-LED devices when driving current varies from 100 mA to 500 mA, respectively. The optical power *P* is defined as the integration of spectrum intensity from 360–830 nm and the luminous flux (LF) is integrated from the spectrum by weighting the human visual function. In detail, the optical power *P* and luminous flux LF read as follows:

(1) $$P=\int_{360\;nm}^{830\;nm}S(\lambda )d\lambda$$

(2) $$LF=683\;lm/W\times \int_{360\;nm}^{830\;nm}V(\lambda )S(\lambda )d\lambda$$

where *S*(*λ*) denotes the spectrum intensity of a LED device while *V*(*λ*) represents the human eye sensitivity function.

As shown in Figure 2a, the SP-RP film and DP-RP configurations show higher optical powers than the RP configuration at all driving currents. In detail, compared with the RP film device, the increasing ratios at 300 mA are 21.55% and 18.03% for SP-RP film and DP-RP film configurations, respectively. As shown in Figure 2b, the luminous fluxes of these three devices increase with the driving current. In terms of the detailed luminous fluxes, the SP-RP film and DP-RP film configurations show higher values (51.82 lm and 55.16 lm) than that of conventional RP film configuration (38.02 lm) at 300 mA. The increasing ratios are 36.28% and 45.1%, respectively.

To further clarify the optical characteristics of the three LED devices, we have measured their spectra at 300 mA, and calculated the blue power *P*_b_ (integration from 360–490 nm), yellow power *P*_y_ (integration from 490–830 nm) and the yellow-blue ratio (YBR). In detail, their expressions can be written as:

(3) $$P_{b}=\int_{360\;nm}^{490\;nm}S(\lambda )d\lambda$$

(4) $$P_{y}=\int_{490\;nm}^{830\;nm}S(\lambda )d\lambda$$

(5) $$YBR=P_{y}/P_{b}$$

To quantify the energy efficiency of three types of RP films, we introduce color conversion efficiency (CCE) to evaluate the blue-to-yellow conversion ability of RP films, which reads as follows:

(6) $$CCE=P_{y}/(P_{b\_ex}-P_{b})$$

where *P*_b_ex_ denotes the optical power of excitation blue light.

As shown in Figure 2c, the spectra distributions change significantly with the coupling of micro-prism films, while their emission peaks are not shifted, which demonstrate that the micro-prism films only affect the emission intensity of yellow phosphor. In detail, the yellow emission powers of the three devices are 82.38 mW, 113.19 mW and 121.79 mW, whereas blue emission powers are 38.08 mW, 33.23 mW and 20.39 mW, respectively, as shown in Figure 2d. The increasing ratios of yellow emission power for the SP-RP film and DP-RP film configurations are 37.4% and 47.84%, respectively, compared with the RP film configuration. The improvements demonstrate that the micro-prism array is effective to enhance the yellow light emission emitted from the phosphor. It is well noted that more yellow emission is produced accompanied by the decreasing blue radiation power of 12.74% and 46.45% for SP-RP film and DP-RP film configurations, respectively. Although the blue emission power is lower for the micro-prism patterned configurations, their total optical power still witnesses gains, compared with the pristine configuration. Based on this, we can anticipate that the backward emission of the RP film should be largely guided upward and further extracted to free space. The detailed analysis will be illustrated later. In addition, the YBR values of the SP-RP film and DP-RP film configurations increase greatly by 57.45% and 176.1%, respectively, in contrast to the RP film configuration as shown in Figure 2d. The YBR enhancement demonstrates that more blue light is converted into yellow emission, which enables a less amount of phosphor to be used when achieving the same CCT. Furthermore, the CCEs of three configurations are calculated to be 32.27%, 43.52% and 44.62%, respectively. The improved CCE clearly indicates that the micro-prism film is conducive to the blue-to-yellow conversion process, agreeing well with the aforementioned light output enhancement.

**Figure 3:**
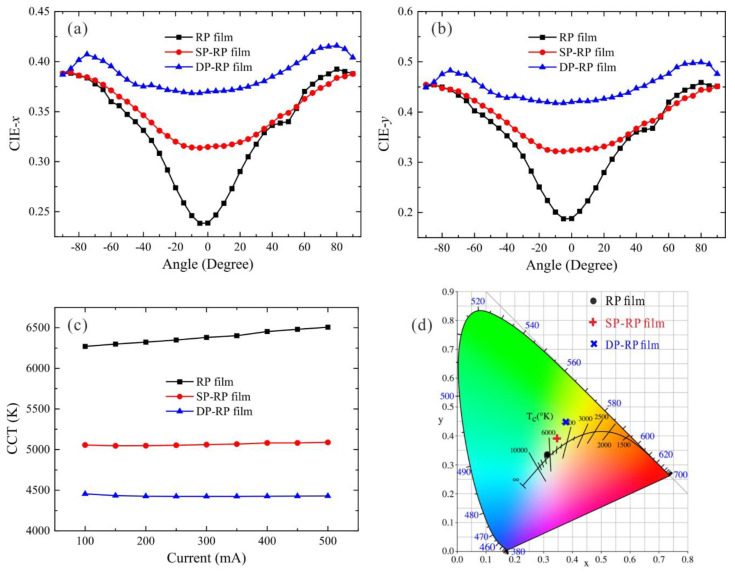
(**a**) Angle-dependent CIE-x and (**b**) CIE-y distributions of three rpc-LED devices at 300 mA measured in the angular range from −90° to 90°; (**c**) current-dependent CCT distributions and (**d**) the chromaticity coordinates in the CIE-1931 diagram of three rpc-LED devices at 300 mA.

To evaluate the CU of three rpc-LED devices, we measured the angle-dependent chromaticity coordinate distributions (CIE-x and CIE-y) from −90° to 90° at 300 mA. The CU is defined as the standard variation of the angular chromaticity coordinates in the measuring angle range. As shown in Figure 3a,b, the angular CIE-x variation and CIE-y variation of RP film configuration are 0.05, 0.088, respectively. For the SP-RP film configuration, those values are 0.027, 0.047, respectively. It is noted that the DP-RP film configuration shows the least chromaticity coordinates variation (CIE-x and CIE-y: 0.016 and 0.027). Compared with the RP film configuration, the SP-RP film and DP-RP film configurations reduce the angular CIE-x variation by 0.023 (46%) and 0.034 (68%), respectively. Meanwhile, they reduce the CIE-y variation by 0.041 (46.59%) and 0.061 (69.32%), respectively. Since a smaller CIE-x or CIE-y variation represents a better CU, the DP-RP film configuration exhibits the best CU among the three LED devices. In addition, the high CCT effect in the central region is significantly suppressed by the integration of micro-prism films, which enables the practical application of such rpc-LED devices.

We note that color stability under varying driving current is also a key characteristic to evaluate the optical quality of pc-LED devices. Therefore, the CCT distributions at different driving currents were measured. As displayed in Figure 3c, the SP-RP film and DP-RP film configurations exhibit much lower CCT values than the pristine configuration. Lower CCT demonstrates that more blue light is converted into yellow emission, which corresponds to the YBR distributions of the three rpc-LED devices. Moreover, the SP-RP film and DP-RP film configurations have a CCT deviation of 42 K and 30 K, respectively, in contrast to the 236 K of the RP film configuration. The decreasing ratios of those two configurations are 82.2% and 87.29%, respectively. The manner in which the phosphor layer is used determines the quality of color mixing in WLEDs, especially at different currents. The SP-RP film and DP-RP film configurations exhibit fewer color deviations because they better use the phosphor layer to maintain almost the same CCT at different currents. Note that the chromaticity coordinates of the three devices are shown in Figure 3d, whose positions are almost on the same line. The chromaticity coordinate shift demonstrates the improvement of phosphor excitation and emission. In addition, it is well noted that the color rendering index (CRI) is also an important characteristic for pc-LEDs, which evaluates the ability of light sources to render the intrinsic color of objects [23]. Herein, the widely used Ra values are adopted as the CRI of pc-LEDs, which are 75, 68 and 64 for the RP film, SP-RP film, and DP-RP film configurations, respectively. It is obvious that after the integration of micro-prism films, the CRI values of LEDs exhibit a gradual decrease, which is reasonable since the increasing YBR ratio leads to the deviation of chromaticity coordinate of LEDs from the Planckian locus in the CIE 1931 chromaticity diagram as shown in Figure 3d. In terms of the color performance of pc-LEDs, one can see that large CCT variation and deteriorating color rendering performance are induced after the integration of micro-prism films, which is a big issue for practical applications. Nevertheless, such issue can be circumvented by well tailoring the phosphor concentration or film thickness to produce acceptable color performance. 

### 3.2. Mechanism of Optical Performance of Remote Phosphor-Converted Light-Emitting Diodes (Rpc-LEDs) Enhanced by the Micro-Prism Films

**Figure 4:**
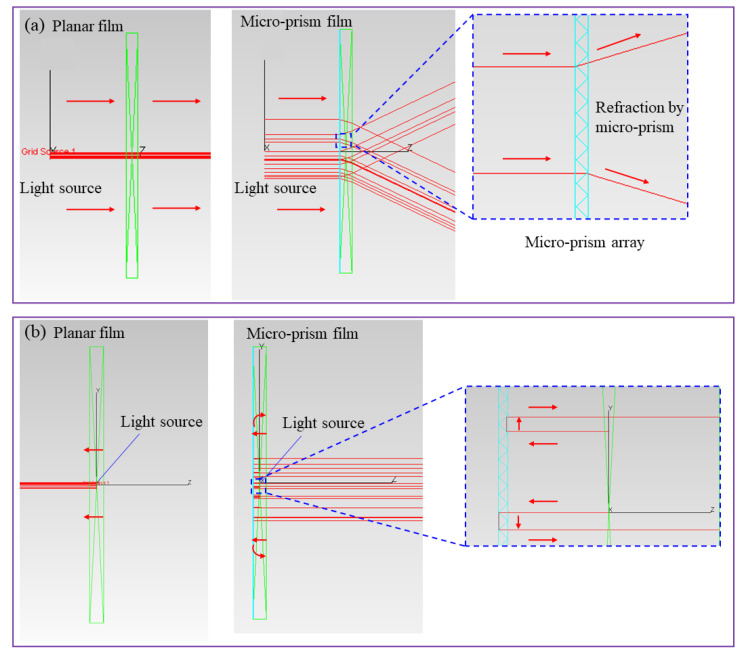
Schematic diagrams of (**a**) light propagation from air to incident surface (left: planar film; right: micro-prism film) and (**b**) light propagation from incident surface to air (left: planar film; right: micro-prism film).

To illustrate the optical mechanism of three LED configurations, ray tracing simulations were conducted to clarify the light transmission path in the planar film and micro-prism film, respectively. As shown in the left panel of Figure 4a, when incident blue light travels from air to planar incident surface, its travelling direction does not change, thus limiting its optical length in the RP film. In contrast, incident blue light is redirected to both sides by the micro-prism array when it enters into the RP layer, as shown in the right panel of Figure 4a. The redirecting mode has two effects: one is elongating the optical length of incident blue light to improve the absorption probability by the YAG phosphor; the other is lowering the central intensity of blue light and further reducing the central CCT. Therefore, the yellow emission power is enhanced and the central CCT is largely reduced after a micro-prism film is integrated on the incident surface. However, this still does not explain the power enhancement of SP-RP film configuration because such a down-converting process of YAG phosphor is accompanied by Stokes power loss. 

Figure 4b can explain the improvement of optical power well. For the conventional RP film, more than half of the emission, including blue and yellow light, is propagating backward [12]. Most of the backward propagating light, including blue and yellow light, enters into the TIR lens and is eventually absorbed by the sleeve and LED substrate, thereby leading to a large power loss for the conventional configuration. With the integration of a micro-prism film on the incident surface of RP film, a large part of downward light can be guided upward, especially for the light at the normal direction. The micro-prism effect is similar to the air gap in reference [24]. Double reflection occurs at the micro-prism interface for blue light and yellow light to reduce the energy loss, which help to improve the optical power. Additionally, part of the guided blue light is absorbed by the phosphor and further converted into yellow emission. Added with the down-converted and extracted yellow emission, the total yellow component witnesses a significant enhancement along with a further decrease of central CCT. Therefore, the SP-RP film configuration achieves improvements in both optical power and luminous flux and a great reduction in the central CCT, compared with the conventional configuration. In addition, since more yellow light transmits backward compared with blue light, yellow light emission witnesses a larger enhancement, which indicates that such optical effect is beneficial for yellow light re-utilization. 

**Figure 5:**
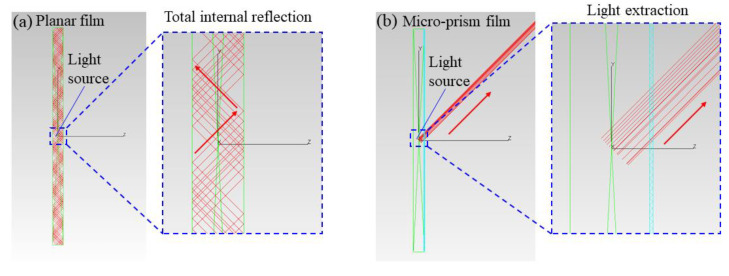
Schematic diagram of light propagation from exit surface to air ((**a**) planar film (**b**) micro-prism film).

Here we discuss the effect of micro-prism film on the exit surface. Without the micro-prism film on the exit surface, the blue light at the normal direction travels directly to air, whereas the blue light is reflected back to the phosphor layer, which can further excite the YAG phosphor to enhance yellow emission (light path is same as that in Figure 4b). Such a trapping effect can bring a large enhancement of yellow emission and also an obvious reduction in the central CCT. It is noted that the micro-prism array also traps the yellow light at the normal direction, which is not favored for light extraction. But for the TIR light (including blue ray and yellow ray), it can be extracted by the micro-prism array. As shown in Figure 5a, a waveguide phenomenon occurs for the total internally reflected light, which leads to a light loss, whereas such trapped light can be extracted outside efficiently by the micro-prism array (as shown in Figure 5b), thereby improving the light extraction efficiency. With the trade-off between light trapping and extraction, the power does not witness a large decrease for the DP-RP film configuration, which is indeed larger than that of the RP film configuration. Since human eyes are more sensitive to yellow emission than blue light, the luminous flux of the DP-RP film configuration still has a further increase on the basis of SP-RP film configuration. Moreover, more produced yellow emission reduces the central CCT, which enhances the CU of the rpc-LED device. Therefore, the DP-RP film configuration still achieves an improvement in the luminous flux at an expense of optical power and a great decrease in the central CCT, compared with the SP-RP film configuration.

## 4. Conclusions

In this work, micro-prism patterned RP film was proposed to enhance both LE and CU of rpc-LEDs simultaneously. The micro-prism films were fabricated by a roll-to-roll process and integrated on the dual sides of a RP film to form a SP-RP film and a DP-RP film, respectively. The micro-prism film on the incident surface of the RP film can guide central light to both sides and extract backward light by double reflection, while the film on the exit surface of the RP film can retain blue light in the RP film to enhance its absorption by phosphor. Experimental results show that the SP-RP film and DP-RP film configurations display increasing ratios of 36.28% and 45.1% in the LE (300 mA), respectively, compared with conventional RP film configuration. As regards the CU, the SP-RP film and DP-RP film configurations reduce angular CIE-x standard variation (300 mA) by 46% and 68%, respectively. Meanwhile, the decreasing ratios are 46.59% and 69.32% for CIE-y variation (300 mA). Moreover, the SP-RP film and DP-RP film configurations show excellent color stability with CCT deviations of only 42 K and 30 K, in contrast to 236 K of the RP film configuration, when driving current varies from 100 mA to 500 mA. Note that large CCT variation and deteriorating CRI are induced after the introduction of micro-prism films, which might be circumvented by optimizing phosphor parameters (i.e., phosphor concentration or film thickness). Since micro-prism films can be easily fabricated by a roll-to-roll process, the micro-prism patterned RP film can be an alternative to a conventional RP layer to enable the practical application of rpc-LEDs.

## Data Availability

The data presented in this study are available on request from the corresponding author.
